# Parenting with mental illness among patients presenting to a teaching hospital in Sri Lanka: Challenges and perceived care needs

**DOI:** 10.1192/j.eurpsy.2021.1920

**Published:** 2021-08-13

**Authors:** Y. Rohanachandra, I. Amarabandu, L. Rohanachandra

**Affiliations:** 1 Department Of Psychiatry, University of Sri Jayewardenepura, Nugegoda, Sri Lanka; 2 Special Care Baby Unit, Colombo South Teaching Hospital, Nugegoda, Sri Lanka

**Keywords:** parenting with mental illness, challanges, impact

## Abstract

**Introduction:**

Parenting with mental illness is associated with parenting difficulties and increased mental health problems in children. Family focused interventions improve child outcomes by 40%. However, such services are not available at present in Sri Lanka.

**Objectives:**

To assess the challenges faced and perceived needs of parents with mental illness in Sri Lanka.

**Methods:**

A cross sectional descriptive study was carried out in the adult psychiatry follow-up clinics in a Teaching Hospital in Sri Lanka. A specifically designed questionnaire was used to collect socio-demographic details, difficulties with parenting and perceived care needs.

**Results:**

Of 385 parents, 67.3% believed their mental illness impacted their parenting. Perceived impact on parenting was higher in younger parents (p<0.01), when the children were younger (p<0.01), had more children under 5 years (p<0.01) and when there was no social support (p<0.01). 67.8% of parents believed their illness impacted their children, with higher impact perceived in parents with children less than 5 years of age (p<0.05) and those with no social support (p<0.01). Although 36.4% had concerns about their children’s emotions or behaviour, only 16.4% were willing to discuss these with their doctor. The parental concerns were significantly higher where the parent was employed (p<0.01), had a longer duration of illness (p<0.01) and when the youngest child was more than 12 years (p<0.01)

**Conclusions:**

Mental illness in parents had a substantial impact on parenting and their children but professionals help was rarely sought. Services aimed at the specific needs of these parents should be developed.

**Disclosure:**

No significant relationships.

